# Supplementary material for: A novel statistical method predicts mutability of the genomic segments of the SARS-CoV-2 virus
Source: QRB Discov. 2021 Dec 13;3:e1. doi: 10.1017/qrd.2021.13 (PMC8795775; doi:10.1017/qrd.2021.13)
Supplement: Supplementary file 1 [file qrdsup.zip › S2633289221000132sup002.pdf]

Supplementary Information

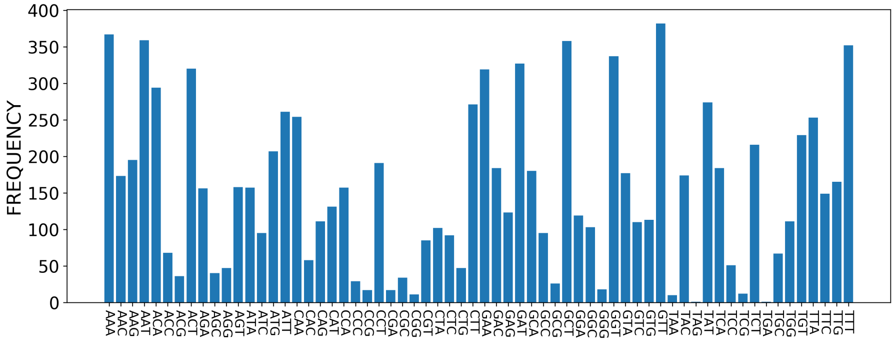

Figure S1: Frequency of occurrence of each codon in the reference SARS-CoV-2 whole genome NC_045512.2.

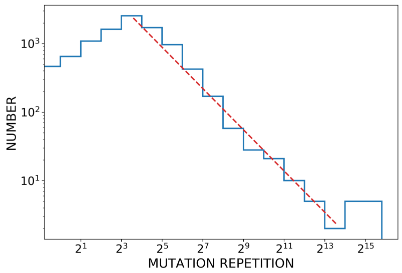

Figure S2: Distribution of total codon mutations along the SARS-CoV-2 genome, according to the GISAID data set. The plot shows the number of positions in the SARS-CoV-2 genome with the number of codon mutations specified along the x-axis. The dashed red line corresponds to a power law function with the form $y \propto x^{-1}$.

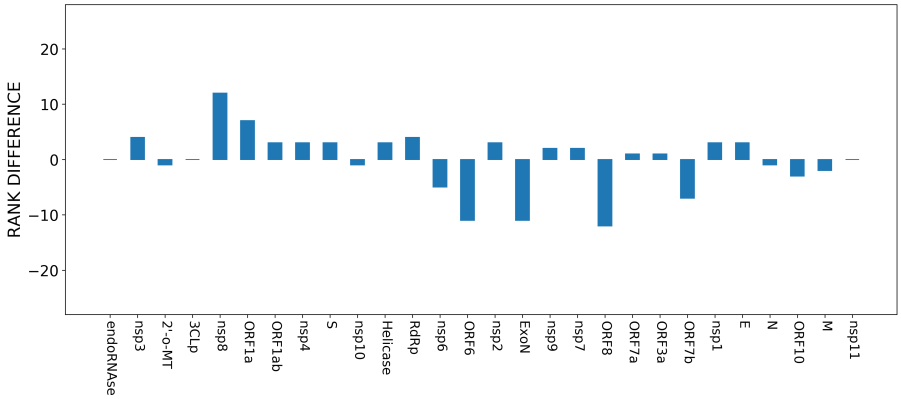

Figure S3: Difference between the rank of the coding regions using the importance value, Eq. (2) and the relative density, Eq. (3). The figure illustrates that the rank difference is less than a few positions for more than half of the coding regions. The coding regions are arranged along the x-axis in order of decreasing importance.

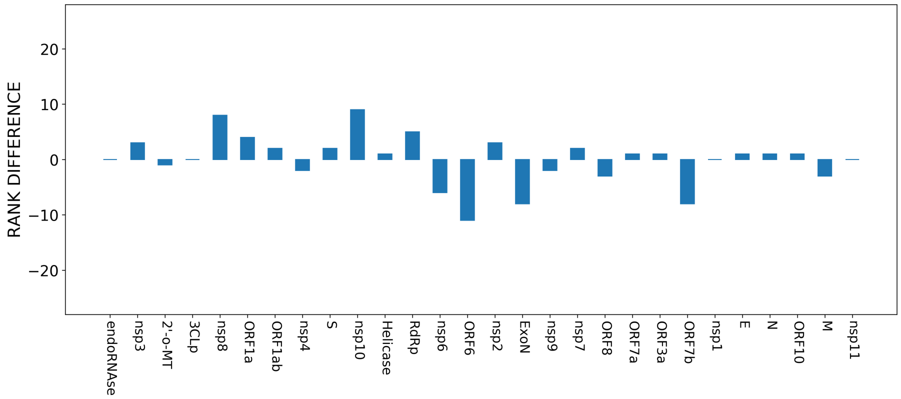

Figure S4: Difference between the rank of the coding regions using the importance value, Eq. (2) and the mutation index. The figure illustrates that the rank difference is less than a few positions for more than half of the coding regions. The coding regions are arranged along the x-axis in order of decreasing importance.
